# Effects of a progressive aquatic resistance exercise program on the biochemical composition and morphology of cartilage in women with mild knee osteoarthritis: protocol for a randomised controlled trial

**DOI:** 10.1186/1471-2474-14-82

**Published:** 2013-03-07

**Authors:** Benjamin Waller, Matti Munukka, Juhani Multanen, Timo Rantalainen, Tapani Pöyhönen, Miika T Nieminen, Ilkka Kiviranta, Hannu Kautiainen, Harri Selänne, Joost Dekker, Sarianna Sipilä, Urho M Kujala, Arja Häkkinen, Ari Heinonen

**Affiliations:** 1 Department of Health Sciences, University of Jyväskylä, Jyväskylä, Finland; 2 Rehabilitation and Pain Unit, Kymenlaakso Central Hospital, Kotka, Finland; 3 Department of Diagnostic Radiology, Oulu University Hospital, Oulu, Finland; 4 Department of Radiology, University of Oulu, Oulu, Finland; 5 Department of Orthopaedics and Traumatology, University of Helsinki and Helsinki University Hospital, Helsinki, Finland; 6 Department of Orthopaedics and Traumatology, Jyväskylä Central Hospital, Jyväskylä, Finland; 7 Unit of Family Practice, Central Finland Central Hospital, Jyväskylä, Finland; 8 LIKES Research Centre, Jyväskylä, Finland; 9 Department of Rehabilitation Medicine, VU University Medical Center, Amsterdam, the Netherlands; 10 Gerontology Research Center, University of Jyväskylä, Jyväskylä, Finland; 11 Department of Physical Medicine and Rehabilitation, Central Finland Central Hospital, Jyväskylä, Finland

**Keywords:** Osteoarthritis, Quantitative MRI, T2 relaxation time, dGEMRIC, Bone, Aquatic exercise

## Abstract

**Background:**

Symptoms associated with osteoarthritis of the knee result in decreased function, loss of working capacity and extensive social and medical costs. There is a need to investigate and develop effective interventions to minimise the impact of and even prevent the progression of osteoarthritis. Aquatic exercise has been shown to be effective at reducing the impact of osteoarthritis. The purpose of this article is to describe the rationale, design and intervention of a study investigating the effect of an aquatic resistance exercise intervention on cartilage in postmenopausal women with mild knee osteoarthritis.

**Methods:**

A minimum of 80 volunteers who meet the inclusion criteria will be recruited from the local population through newspaper advertisements. Following initial assessment volunteers will be randomised into two groups. The intervention group will participate in a progressive aquatic resistance exercise program of 1-hour duration 3 times a week for four months. The control group will be asked to maintain normal care during this period. Primary outcome measure for this study is the biochemical composition of knee cartilage measured using quantitative magnetic resonance imaging; T2 relaxation time and delayed gadolinium-enhanced magnetic resonance imaging techniques. In addition, knee cartilage morphology as regional cartilage thickness will be studied. Secondary outcomes include measures of body composition and bone traits using dual energy x-ray absorptiometry and peripheral quantitative computed tomography, pain, function using questionnaires and physical performance tests and quality of life. Measurements will be performed at baseline, after the 4-month intervention period and at one year follow up.

**Discussion:**

This randomised controlled trial will investigate the effect a progressive aquatic resistance exercise program has on the biochemical composition of cartilage in post-menopausal women with mild knee osteoarthritis. This is the first study to investigate what impact aquatic exercise has on human articular cartilage. In addition it will investigate the effect aquatic exercise has on physical function, pain, bone and body composition and quality of life. The results of this study will help optimise the prescription of aquatic exercise to persons with mild knee osteoarthritis.

**Trial Registration:**

ISRCTN65346593

## Background

Osteoarthritis (OA) of the lower limb is a leading cause of decreased function and quality of life [1]. It has been estimated that the prevalence of symptomatic OA of the knee is between 7-33% with an increase in prevalence with age and is the most common site of symptomatic OA [2-6]. Early signs of OA in articular cartilage, which is constituent for the initiation and progression of OA, are characterised with loss of proteoglycans, breakdown of the collagen matrix and increased water content [7]. As the disease progresses there is fibrillation of the cartilage, changes in the subchondral bone, formation of osteophytes and thickening of the synovium [8-11] and as such OA is considered a whole joint disease. These modifications within the joint lead to the gradual development of clinical symptoms such as stiffness, decreased range of motion and pain [12] which cause a decrease in joint proprioception [13] and inhibits muscle activation [14,15] leading to a decrease in activity. This disuse results in a lowering of aerobic capacity, muscle strength and muscle mass and ultimately a decrease in functional capacity and increased dependence [16,17]. Additionally, reduced muscle strength is a risk factor for future pain [17], self-reported knee instability [18] and increased risk of falling [19]. These in combination cause the extensive social and medical costs to society as a direct or indirect result of OA.

Although there is no known cure for OA the disease-related factors such as impaired muscle function and reduced aerobic fitness can be improved and maintained with therapeutic exercise [20,21]. Previous systematic reviews have demonstrated that exercise has positive effects on pain and function for people with symptomatic OA of the knee [21-23] and is recommended as one of the primary non-pharmaceutical treatment modalities in current OA guidelines [24-29]. Exercising in water is also strongly recommended in these guidelines. There is evidence to suggest that therapeutic aquatic exercise has a short term positive effect on pain and function in persons with OA of knee and/or hip similar to that of land training [30,31]. There is good evidence to support the use of strength exercises in the management of symptoms resulting from OA [32] however, there is conflicting evidence that therapeutic aquatic exercises can improve strength of lower limb muscles in persons with OA [33-40]. It is thought that the benefits from aquatic exercise are primarily a result of the decreased effects of gravity. Buoyancy reduces compressive and shear forces on joints and thus offers a comfortable training medium for patients with OA [41].

Previously, one restriction in OA research was the lack of non-invasive *in vivo* techniques to quantify the structure and acute changes in cartilage. Advances in magnetic resonance imaging (MRI) have made mapping of the articular cartilage and loading related changes possible [42]. The “delayed Gadolinium Enhanced MRI of Cartilage” (dGEMRIC) technique utilizes a paramagnetic contrast agent gadolinium (Gd-DTPA^2-^) to detect early reduction of glycosaminoglycan (GAG) from the matrix, a phenomenon considered to represent the onset of the degenerative process of cartilage [43]. Measurement of T2 relaxation time, sensitive to degeneration of tissue collagen and the orientation of collagen fibres in the extracellular matrix, has been developed to detect early degeneration or senescent changes of cartilage [44,45]. In addition, the assessment of morphological properties from three-dimensional MRI measurements enables assessment of tissue changes at a macroscopic scale [46] which have been found to be reliable, responsive and valid methods for mapping the volumetric data of articular cartilage [47-49].

There is still a lack of evidence that human cartilage can adapt to mechanical loading in a similar way to other tissues such as bone and muscle. Animal studies have suggested that physical exercise can improve tissue integrity by increasing the GAG content and indentation stiffness in load bearing cartilage [50,51]. In a cross-sectional study Tiderius et al. [52] concluded, based on dGEMRIC measurements, that GAG content was higher in regularly exercising individuals than in sedentary subjects. Additionally, observations by Teichtahl et al. [53] suggest that vigorous physical activity is associated with a reduced rate of patella cartilage volume loss in asymptomatic subjects. To date, only one randomised intervention study investigating the direct effect of exercise on biochemical composition of human cartilage [54] has been published. Roos et al. [54] reported a positive effect of a moderate four months exercise on the GAG content, measured with dGEMRIC in subjects with high risk of knee OA. Another study by Cotofana et al. [55] provides no evidence that a 3-month exercise intervention in untrained middle-aged women can significantly alter cartilage morphology in the knee joint. Furthermore, the optimal type or intensity of exercise for improvement in cartilage is not known and longitudinal effects of training are needed to determine the exercise response once OA is established. In particular, there are no studies investigating the effect non-impact training such as therapeutic aquatic exercise has on the structures related to and progression of OA in the knee joint.

Therefore we plan to investigate the effects of an intensive aquatic resistance exercise program on the biochemical composition and morphology of the knee cartilage as well as its effect on physical function in postmenopausal women with mild knee osteoarthritis. In addition, we plan to discover if the possible benefits of exercise on cartilage, symptoms and physical function can be maintained one year after training period.

The purpose of this article is to describe the rationale, design and intervention of a study investigating the effect an aquatic resistance exercise intervention has on the cartilage in postmenopausal women with mild knee osteoarthritis.

## Methods and design

### Study design

The design of this study will be a 4-month randomised controlled exercise intervention study (RCT) with a 16 month follow up (Trial registration: ISRCTN65346593). After baseline measurements the voluntary participants will be randomly assigned into the two arms of the study, an aquatic resistance strength training group and a control group. All the outcome measurements will be performed at baseline, after the 4-month intervention and at follow up 12 months after cessation of training.

### Participants and selection criteria

Volunteer postmenopausal women, between the ages of 60–68 year-old, will be recruited through a series of local newspaper advertisements and will be gathered from the county of Central Finland which has a population of approximately 275 000. Inclusion eligibility, (see below), will be initially assessed using a structured telephone interview. The telephone questionnaire includes questions concerning degree of knee pain, current level of physical activity and past medical history.

Suitable participants will be taken forward and they will undergo weight bearing x-ray imaging of both knees. An experienced radiologist and orthopaedic physician will assess the images grading the degree of OA in the tibiofemoral and patellofemoral joints using the Kellgren-Lawrence grading (K/L 0-IV) [56]. Those participants who have a KL score of I (possible osteophytes) or II (definite osteophytes, possible joint space narrowing), will be included in the next stage of eligibility assessment and undergo a medical and physiotherapy screening. At this point any possible physical or medical limitations to full participation in the intervention will be assessed e.g. severely restricted joint range of movement (ROM), excessive laxity of knee joint, possible physical disabilities and abnormalities found from resting echocardiogram.

Subjects will be excluded if they have at least one of the following criteria; BMI > 34, resting pain in knee VAS > 50/100, known loose particles in knee joint, acute inflammation in knee joint, knee intra-articular steroid injection in previous 3 months or oral steroid medication treatment in the previous 12 months, undergoing treatment for osteoporosis or T-score for femoral neck bone mineral density (BMD, g/cm^2^) lower than −2.5 i.e. indicating osteoporosis as measured with DXA [57-59], previous cancer or radiotherapy, suffer from type I or II diabetes, cardiac disease, diagnosed rheumatic disease (other than OA), undergone surgical procedure to knee (excluding menisectomy or arthroscopy if over 12 months ago) or joint replacement surgery in lower limbs.

**Figure 1:**
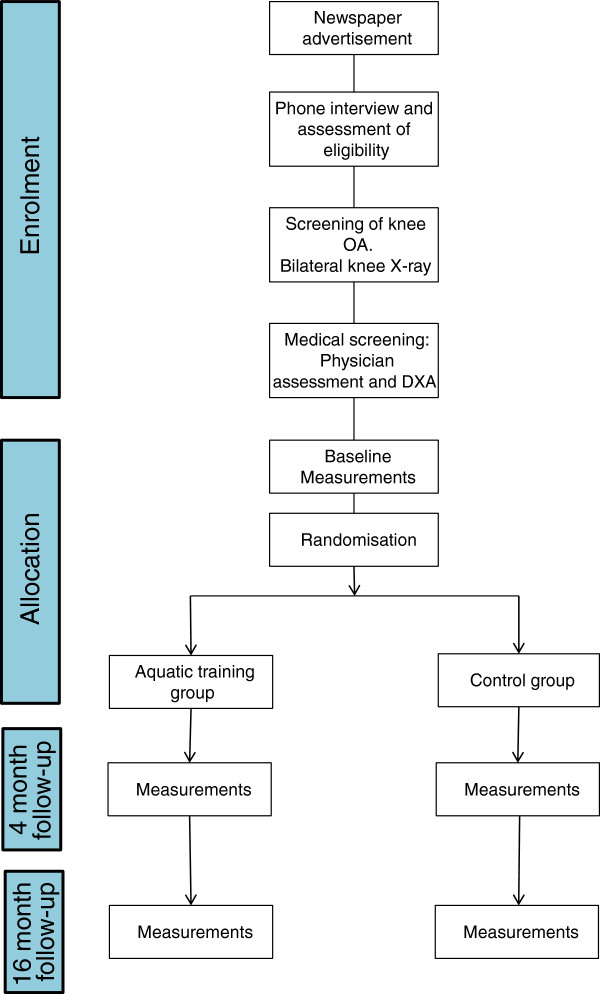
Flow chart showing enrolment, allocation and follow-up measurements.

Additional exclusion criteria are problems that would prevent MRI imaging, including electronic or magnetic implants e.g. pace maker, metal within body e.g. internal bone fixations, artificial aortic heart valve, metal particles in eyes, large tattoos on lower limb, claustrophobia or possible allergy to the contrast medium. Further, fasting blood samples will be taken to analyse Krea to ensure kidney function for normal removal of contrast medium from the body. All those participants fulfilling all the inclusion criteria will be included into the study and undergo the baseline measurements. Figure 1 shows the flow chart describing the selection and measurement procedure for the whole study.

### Sample size

The sample size and power calculations have been estimated for the primary end points of this study, i.e. the dGEMRIC and T2 variables. Based on data from Roos et al. [54] and Tiderius et al. [55] it is estimated that 30 subjects are needed, at 80% power, to detect a mean ± SD difference of 40 ± 40 msec in the dGEMRIC between groups [54]. It is estimated that dropout rate will be about 20% at the 16 months follow up, consequently at least 70 subjects will need to be recruited.

### Randomisation and blinding

The subjects will be randomly allocated into either of the two arms of the study by an external statistician blinded for the intervention and study participants and will only be provided with a randomisation number for each participant and severity of OA in knee according to x-ray classification. A computer generated block randomisation of size of ten, stratified according to Kellgren-Lawrence grading 1 and 2, will be used to ensure equal distribution of severity of OA within each group and equal group size.

As with all exercise intervention studies blinding of the subject from the intervention is not possible. Researchers (BW, MM, AH) will be blinded to the allocation of groups as well as blinded from the interventions and measurement except for pQCT (MM) and DXA (BW) measurements. Due to practical limitations the physical therapists providing the intervention will also be performing the physical performance measurements. All statistical analyses will be completed by a statistician (HK), who is blinded to the participants and measurements.

### Primary outcomes

This research project will have two primary outcome measures. Delayed gadolinium-enhance magnetic resonance imaging of cartilage (dGEMRIC), sensitive to the distribution of GAG, will be used to evaluate the biochemical composition of cartilage. Arrangement of collagen and hydration state of the cartilage will be measured using T2 relaxation time mapping. Furthermore, knee cartilage morphology as a regional cartilage thickness will be analysed from the weight bearing area of tibiofemoral and patellofemoral cartilages. The dGEMRIC method has been validated in several *in vitro* studies [60-62] and it had been applied in several *in vivo* studies [43,52,63-71]. Also, T2 relaxation time method has been histologically validated *in vitro*[72], and it has been applied in several human studies to assess chondral repair [69,73-76].

### MRI protocols

Prior to imaging, the subject will be advised to restrain from any strenuous physical activity during the 48 hours prior to the measurements to minimise possible transient changes in knee cartilage volume and composition. Subjects will be imaged at the same time of the day to avoid possible diurnal variation at the follow-up measurements. The participants will be imaged lying supine with knee to be imaged in slight flexion, stabilized in a leg holder and a custom made inflatable cushion. The cushion has been specifically designed to stabilize the patella without causing any compression of the patellofemoral joint. The imaging session will last in total 3 hours and will include initially a standard clinical MRI series and T2 relaxation time followed by a dGEMRIC series.

T2 mapping will be performed using a sagittal multi-slice multi-echo fast spin echo sequence (field of view (FOV) 140 mm, acquisition matrix 256 x 256, repetition time (TR) 2090 ms, eight echo times (TE) between 13 and 104 ms, echo train length (ETL) 8, slice thickness 3 mm). The slices will be positioned perpendicular to a line tangential to the posterior femoral condyles in the axial scout view. Two slices, each covering the central region of the medial and lateral condyles, will be analysed.

For the dGEMRIC series, immediately after the clinical and T2 imaging a double dose of Gd-DTPA^2-^ (Magnevist, Schering, Berlin) will be administered intravenously i.e., 0.4 ml/kg (0.2 mM/kg). At baseline, post intervention and 16 month follow up the amount of contrast administered will be corrected for body weight. It is felt this is appropriate because of the expected changes in body composition as a result of the intensive exercise intervention. In order to enhance the delivery of contrast agent into the knee cartilage, following administration of Gd-DTPA^2-^ the subject will be instructed to perform 5 minutes of flexion-extension exercises in a sitting position without resistance, 5 minutes of walking on a flat surface and 10 gentle deep squats. Exactly ninety minutes after the injection, T1 mapping in the presence of Gd-DTPA^2-^ (dGEMRIC) will be performed in the sagittal plane using a single slice inversion recovery fast-spin echo sequence (FOV = 14 cm, matrix 256 x 256, TR = 1800 ms, TE = 13 ms, six inversion times (TI) between 50 and 1600 ms, slice thickness 3 mm). The slice positioning will be copied from the T2 relaxation time mapping sequence, and the number of the slices in the correct orientation is reduced to one. The remaining slice is then positioned at the centre of the medial and lateral condyles as viewed on the axial scout image. The subject will be positioned into an identical position as for the first MRI imaging. For both the MRI images and pQCT measurements the knee with highest degree OA, as measured by the radiographic Kellgren-Lawrence scale, will be imaged. In the cases were both knee have identical KL score the right knee will be imaged.

### Segmentation

Weight bearing cartilage regions of interest (ROIs) from single sagittal slices at the centre of the medial and lateral tibial and femoral condyles will be segmented using a semi-automated in-house MATLAB application (Mathworks, Inc. Natick, MA, USA). dGEMRIC indices will be corrected for BMI [77]. In this research team the *in vivo* precision of dGEMRIC for full thickness cartilage in different ROIs ranges from 5% to 7% [78]. The inter-observer precision of T2 in different locations is on average 5% [79]. For quality assurance purposes, a set of phantom samples containing certain concentrations of agarose and nickel nitrate to modulate their T1 and T2 relaxation times will be imaged following the study protocol prior to baseline and follow-up measurement sessions to assess possible drift.

### Secondary outcomes

#### Properties of bone and body composition

##### Peripheral quantitative computed tomography (pQCT)

The bone properties of the distal radius and mid and distal tibia will be measured using a pQCT device (XCT-2000; Stratec Medizintechnik, Pforzhem, Germany). A 2-mm-thick single tomographic slice with pixel size 0.59 mm in plane resolution will be taken at 5% and 55% of the length of the tibia proximal to the distal end of the tibia. Lower leg length is defined as the distance between the medial condyle of tibia and medial malleolus. Selection of lower limb to be imaged will be based on the same principles as the MRI scan. The forearm slice will be taken at 4% of ulna length proximal to the distal endplate of ulna. Length of ulna is defined as the distance between olecranon process and the midline of lateral aspect of distal ulna. In all cases right upper limb will be scanned except when subjects had suffered from fracture of either right ulna or radius. The analysis of the pQCT images will be performed with the density distribution plug-in [80] of the BoneJ (http://bonej.org/densitydistribution) [81] ImageJ (http://rsbweb.nih.gov/ij/download.html) plug-in. Compressive bone strength index (BSI_d_, g^2^/cm^4^), bone mineral content (BMC), total and trabecular density (ToD and TrD, mg/cm^3^) and total and trabecular area (ToA and TrA, mm^2^) will be analysed from the shaft slices. The pQCT device is calibrated daily using a standard phantom provided by the manufacturer and coefficient of variation (CV) for these protocols in our laboratory has been measured to range between 1.5-3.4% for the reported variables [82].

#### Dual-energy X-ray absorptiometry (DXA)

DXA (Lunar Prodigy; GE Lunar Healthcare, Madison, WI, USA) will be used to assess body composition and bone traits. Body composition analyses will be carried out using enCORE software (ENcore 2011, version 13.60.033). Using manufacturers software and protocols total body fat and lean body mass will be measured. *In vivo* precision of these measurements has been reported to be CV 1.3-2,2% [83]. Both proximal femur and Lumbar spine (L2-4) areal bone mineral density (aBMD, g/cm^2^) and bone mineral content (BMC, g) will be scanned. Cross sectional geometry of the femoral neck will be analysed using advanced hip structure analysis (AHA) as per manufacturer’s software. This will include femoral neck hip axis length (HAL, mm), cross sectional area (CSA, mm^2^), cross sectional moment of inertia (CSMI, mm^4^) and femoral neck strength index (FSI, mm^3^) [84-86]. *In vivo* repeatability, CV, of these methods has been reported as 2.3% for CSA [87].

#### Questionnaires

##### Health status

General health and habitual physical activity at baseline will be assessed by a questionnaire devised by the research group. This health questionnaire addresses medical conditions, current medications, years of menopausal hormone therapy, history of fractures and current leisure time physical activity. Throughout the entire follow up period all subjects will be asked to report their daily amount of analgesia taken to manage their knee pain. Space will be provided in the physical activity diary for ease of recording.

#### Impact of osteoarthritis of the knee

Self-assessed impact of osteoarthritis on functioning will be measured using two questionnaires, the Western Ontario and McMaster Universities Osteoarthritis Index (WOMAC) [88] and the knee injury and osteoarthritis outcome score (KOOS) [89]. The visual analogue version (VAS) of the WOMAC (0-100 mm) will be used with a range of scores of 0–2400. This questionnaire has 24 questions and is divided into three domains; pain (score ranging from 0–500), stiffness (0–200) and function (0–1700). A higher score indicates more disability. The internal consistence (Cronbach’s alpha) for the VAS version is 0.7-0.91 and test-retest (ICC) coefficient is 0.95 for pain, 0.90 for stiffness and 0.92 for function ([90]). A likert version of the KOOS will be used with each response being scored 0–4. The questionnaire has 5 domains: pain (9 questions), other symptoms (7 questions), activities of daily living (16 questions), sport and recreation (5 questions) and knee related quality of life (4 questions). Score for global and domains scores are transformed into a score 0–100 with a score of 0 indicating extreme knee problems and 100 no knee problems. The internal consistency for the KOOS is 0.86-0.96 and test-rest (ICC) is (0.67-0.95) [91]. Reliability of the Finnish language version of both WOMAC and KOOS has been shown to be similar to that of the English language version [92].

#### Quality of life

Self-assessed quality of life will be measured using the RAND-36-Item short form healthy survey instrument [93] this questionnaire is identical in wording to the short form 36 questionnaire (SF-36) but summation of final scores is different. It contains 8 domains: physical functioning (10 items), role limitations due to physical health problems (4 items), role limitations due to emotional problems (3 items), energy/fatigue (4 items), emotional well-being (5 items), social functioning (2 items), pain (2 items), and general health (5 items). Global and individual domains will be re-scored and given values of 0–100 with higher scores indicating a more favourable health state. The scores will also be divided into two summary measure: the physical component summary score (PCS) and the mental component summary score (MCS). The dimensions physical functioning, role limitation due to physical health problems, body pain and general health form the PCS and mental health, energy/fatigue, social functioning and role limitations due to emotional problems form the MCS. In a Finnish standardization population sample aged 18–79 years the homogeneity, i.e., the mean of the item inter-correlations of the Scale, was 0.63 and Cronbach alpha 0.94 [94].

#### Physical performance measures

##### Muscle strength

Maximal isometric knee flexion and extension strength of both legs, as well as grip strength of dominant hand, will be measured using an adjustable dynamometer chair (Good strength; Metitur Ltd, Jyväskylä Finland). The best result from 3 contractions will be used and recorded in newtons (N). In our laboratory, the precision of the test is 6% for knee extension and 9% for knee flexion [95].

#### Muscle power

Single leg extension power will be measured using Nottingham power rig (University of Nottingham Medical School, Nottingham, UK) which has been tested for reliability and has a test retest co-efficient of variation (CV of 9.4%) [96] and in our laboratory the CV is 8% [97].

In addition lower limb power function will be determined by a maximal counter movement jump (CMJ) measured using a custom made force plate (University of Jyväskylä, Finland). This test is a measure of neuromuscular function. Jumping force, vertical ground reaction forces, power, impulse and jump height will be calculated. Data is collected at a sampling frequency of 500 Hz [98].

#### Aerobic fitness

Maximal aerobic power VO_2_ max will be estimated using the UKK 2 km walk test (UKK Institute, Tampere, Finland). This test requires the subject to walk 2 km as quickly as possible with a target of 80% maximal heart rate [99]. VO_2_ max is estimated using walking time, body mass index (BMI), age and heart rate at end of test. The heart rate will be measured by a portable heart-rate monitor (Polar F6, Polar Electro Ltd, Kempele, Finland). It is a feasible test for estimating V02 max [100] and sensitive to changes [101]. Its validity has also been tested with correlation coefficient of 0.69-0.77 [102].

#### Static balance

Static balance ability will be assessed using a force platform device (Goodbalance, Metitur Ltd, Jyväskylä Finland) which is validated and reliable method measuring body sway in different standing positions [103,104]. Balance will be measured in feet side-by-side eyes open and eyes closed and single leg stance [105].

#### Agility

Agility will be assessed with a standardised figure-of-eight running test consisting of two laps around two cones placed 10 meters apart in a figure of eight [106-108]. Time (in seconds) taken to complete the task will be measured using a photocell. This test has shown to be effective at detecting decreased motor performance (area under curve 0.86) additionally it has been shown to be a very sensitive (73.5%) and specific (86.1%) tool for measuring agility [109].

#### Gait

Spatial and temporal parameters of gait will be measured using the GAITRite® walkway (CIR systems, inc. Clifton, NJ 070872) [110]. This consists of a 577 cm long and 88.5 cm wide matt with 13,824 sensors placed on 1.27 cm in a grid. The collection frequency of the matt is 80Hz. The data is transferred by lead to a computer and is analysed using GAITRite 3.6b software. This technique has been validated with different populations [111,112] and found to be a reliable [112,113] instrument to measure spatial and temporal parameters of gait.

#### Daily physical activity

During both the intervention period (0–4 months) and the follow up period (5–16 months) daily physical activity of every subject (excluding pool training) will be recorded using a leisure time physical activity diary. The diary is completed daily and each activity, duration and intensity (1 = low, 2 = moderate or 3 = hard) is recorded. From this data MET-hours per week will be calculated [114,115]. In addition, during the intervention period each subjects’ daily activity will be measured for 3 days using a heart rate monitor (F6 Polar, Polar Oy, Finland), accelerometers (Hookie AM 20, Traxmeet, Finland) and hourly physical activity diaries.

#### Intervention

Those subjects randomised into the intervention group will participate in 1 hour of aquatic resistance training, three times a week for 4 months, totally 48 training sessions. The intervention will be completed in small groups of 6–8 subjects in a pool heated to 32 degrees with depths 1.3-1.5 m. Aquatic steps will be used to ensure that all subjects will complete the standing exercises at a depth level approximately to their xiphoid bone ±5 cm ensuring weight bearing on the supporting leg of 25-50% of own body weight [41].

**Figure 2:**
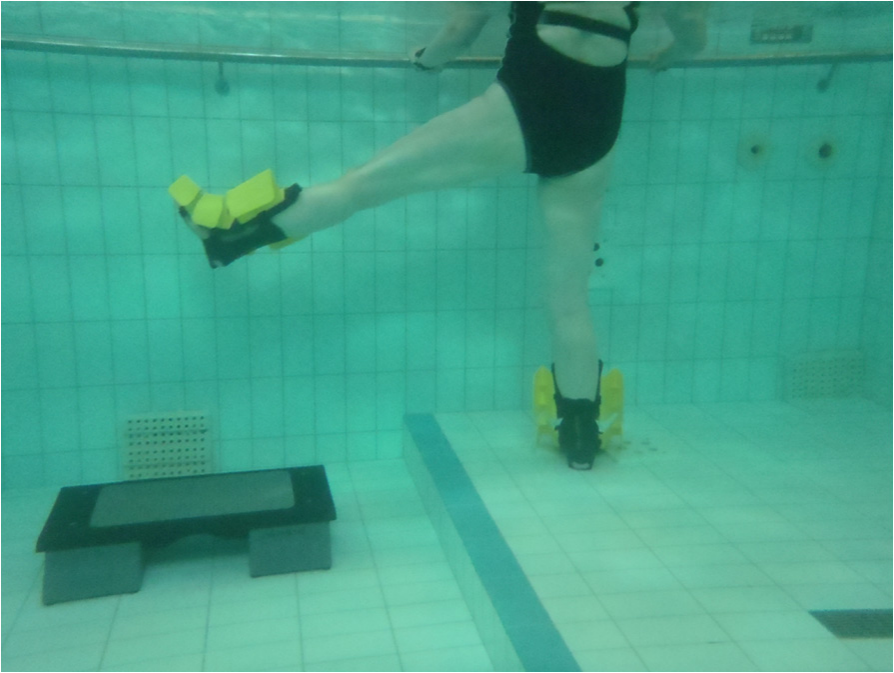
Hip abduction/adduction in standing.

**Figure 3:**
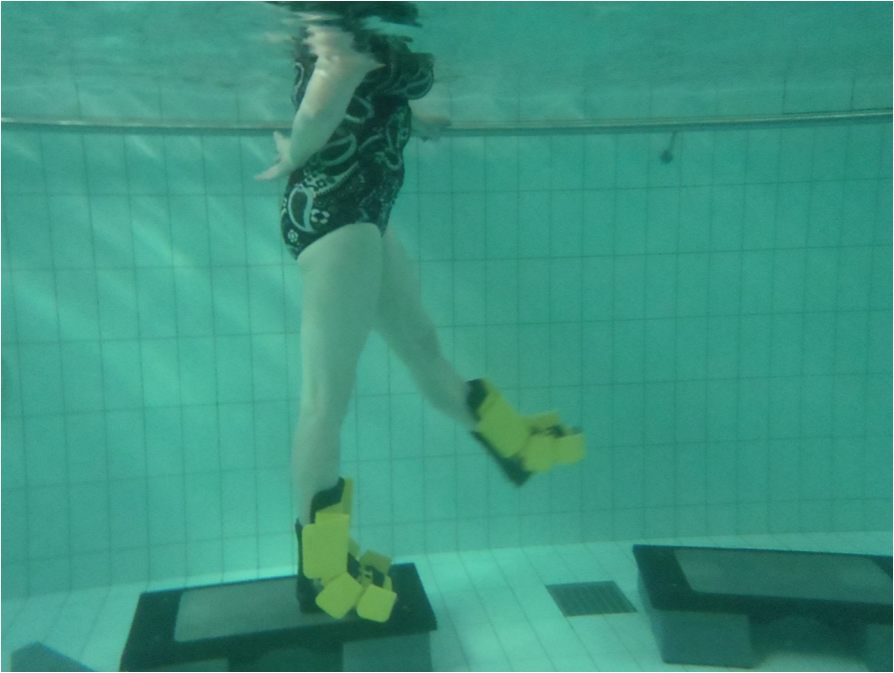
Hip flexion/extension in standing.

**Figure 4:**
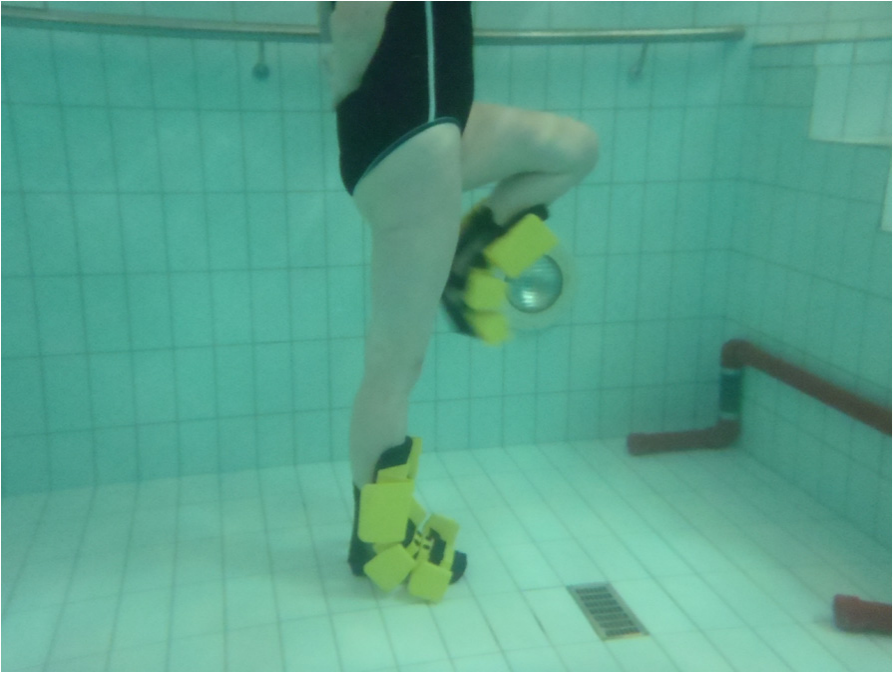
Knee flexion/extension in standing.

**Figure 5:**
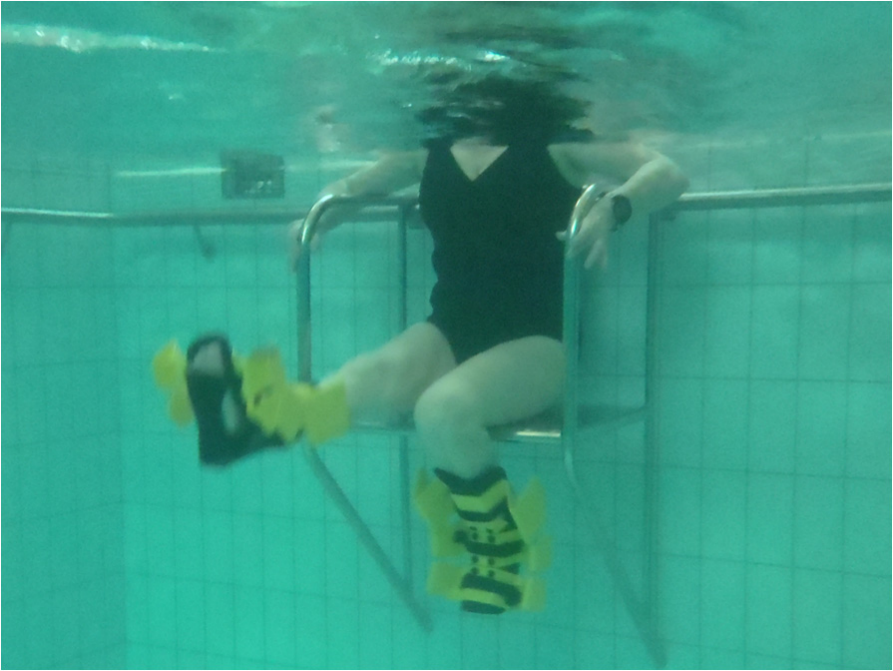
Knee flexion/extension in sitting.

**Figure 6:**
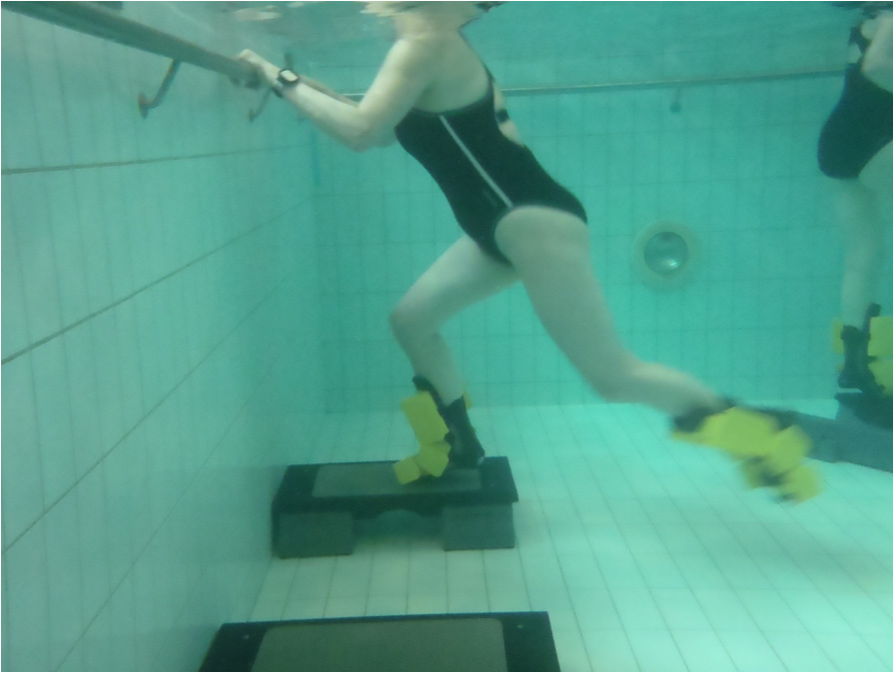
Kickback (reverse lunge).

Each training session will last approximately 1 hour. The session will consist of three distinct parts; the warm-up (15 minutes), lower limb strengthening program (35 minutes) and cool down (10 minutes), a full description of exercises can be found from Table 1 and Figures 2, 3, 4, 5, 6. Warm up and cool down was planned by a physiotherapist with over 10 years of aquatic therapy experience with patients suffering from musculoskeletal problems (BW), the same therapist will ensure that quality of movement and intensity of the intervention is maintained throughout the training by reviewing the heart rate and perceived exertion by BORG 6–20 scale [116] which are collected after every training session immediately after the main set before the cool down. All sessions will be supervised by 2 experienced physiotherapists, who had been trained to instruct these aquatic programmes and accredited for life-saving before the trial began.

**Table 1:** Description of exercises included in the intervention

**Warm-up (10-15 minutes)**	**Strength training (35 minutes)**	**Cool down (10 minutes)**
1. Standing hip flexion/extension	1. Standing hip flexion/extension (Figure 3): Standing side on to wall and stand on leg furthest away from wall. Swing leg nearest wall forwards and backwards as fast as possible using full range of motion. Keep knee of moving leg in full extension and ankle in dorsiflexion. Maintain spinal neutral at all times.	3-5 minutes of walking and supported cycling against wall
2. Standing hip abduction/Abduction	2. Hip adduction/abduction (Figure 2): Stand on right leg with knee fully extended. Keeping knee straight and ankle in dorsiflexion swing left leg from side to side as fast as possible using full range of motion. Left leg crosses over in front of right leg. Particular attention is paid to maintaining a neutral pelvic and spinal position.	Stretches, 30 seconds stretch for each side.
3. Seated bilateral knee flexion/extension.	3. Seated knee flexion/extension (Figure 5): Sitting on chair keeping back of legs fixed against seat, alternately flex and extend both knees as fast as possible using full range of motion.	1. Hip flexors (Iliopsoas)
4. Calf raises on edge of step (weeks 1-8 double leg, weeks 9-16 single leg)	4. Standing knee flexion/extension (Figure 4): Standing side on to wall and transfer weight on to leg furthest away from wall. Lift leg nearest wall straight up in front stopping just before any stretch sensation is felt in the posterior aspect of thigh. Keeping thigh still, flex and extend knee as fast as possible using full range of motion.	2. Gluteus maximus
5. Balance beam (EWAC, Netherlands) walking forwards and backwards (weeks 1-6 without arms, weeks 6-12 carry tray with ball on, 13-16 same eyes closed)	5. Kickback (reverse lunge) (Figure 6): Standing on edge of step board so that moving leg can be swung down next to it. Starting position is with supporting leg in full extend and other hip and knee is in flexion up near wall. Leg is explosively straightened down towards bottom of pool and then kicked backwards with leg straight as far as possible. During this movement supporting leg is flexed but plantar aspect of foot is kept firmly pressed against step.	3. Quadriceps
6. Standing abdominals (either pushing and pull frisbee, trunk rotation with frisbee or rowing with aquatic rolling pin), weeks 1-8 double leg stance, week 9-16 single leg).		4. Hamstrings
7. Abdominal with feet in frisbee against wall (figure of 8, circles 30 second each direction)		5. Iliotibial band
8. Hurdles (EWAC Netherlands), weeks 1-6 stepping over hurdles, weeks 6-12 double leg jumps forwards and backwards over 30 cm hurdle, 13-16 single leg jumps forwards over 30 cm high hurdle)		6. Adductors
9. Weeks 1-6 scissor jumps, weeks 6-12 jumping over 30 cm hurdle sideways, weeks, 13-16 single leg sideways jumping over 30 cm hurdle.		7. Gastrocnemius
10. Dynamic balance ½ of the group jog/run around other ½ of group who are trying to maintain balance.		

The warm-up consists of 10 different movements to increase active ROM of all joints and enhance neuromuscular activation. Each movement will be completed for 1 minute (30 seconds per leg when alternating leg) with a 15 second rest period. Order of movements will be altered for each session randomly to maximise neuromuscular stimulation and prevent staleness as well as to maintain subjects’ interest.

The strength training section consists of 5 exercises which have been thoroughly researched for both their effect on muscle activation [117,118] and effect on muscle strength and physical functioning [119-121]. Focus will be on performing each movement as fast as possible through full ROM. During all standing exercises emphasis will be made on maintaining the lumbar spine in a neutral position thus avoiding excessive loading on the spine and to encourage activation of trunk muscles during exercises. The progression of the exercise program will be ensured by using resistance boots of different sizes and by varying the duration of sets. Table 2 shows the different durations of each set and targeted amount of repetitions per set for each stage of the intervention. Each leg will be trained before resting e.g. 45 seconds left leg, 45 seconds right leg and 30 second rest.

**Table 2:** Intensity and progression of each program for phase’s I-IV of aquatic exercises program

**Weeks**	**Resistance type**	**Sets**	**Repetitions per set**	**Time (sec)**	**Recovery (sec)**	Target PRE*	**Total time (mins)**	**Total No. reps**
1-2	Barefoot	3	25-30	45	30	14-15	30	750-900
3-5 (alternating)	Small	3	20-25	45	30	15-16	30	600-750
	Small	3	12 to 15	30	45	16-17	26	288-360
6-8 and 12	Small/Large	3	14-20	45	30	16-17	30	420-600
9-11 and 13–16 (alternating)	Large	3	14-20	45	30	16-18	30	420-600
	Large	3	12 to 15	30	45	16-18	26	288-360

*PRE = perceived rate of exertion (BORG 6–20).

Weeks 1–2 is an introductory period to allow subjects to become familiar with the movements with sets of 45 seconds duration per leg per set with no resistance i.e. barefoot. Weeks 3–5 will consist of alternate trainings of 30 or 45 seconds with small fins (THERABAND PRODUCTS, The Hygienic Corporation, Akron, OH 44310 USA). Weeks 6–8 will be 3 week period with 45 seconds of work alternating sessions with small aquafins and large resistance boots (Hydro-Tone hydro-boots, Hydro-Tone Fitness Systems, Inc. Orange, CA 92865–2760, USA). Weeks 9–11 and 13–16 will consist of alternate trainings with work of 30 and 45 seconds with large boots. Week 12 will consist of one session barefooted, one with small fins and one with large boots, work duration will be 45 seconds per set. The frontal area of aquafin resistance fins is 0.0181 m^2^ and that of the large resistance boots 0.075 m^2^. In a previous study the drag experienced during seated aquatic knee flexion/extension exercises in healthy women was triple with the large boots compared to the barefoot condition. Additionally, a significant increase in EMG activity was seen with the large boots compared to no boots [117,122].

Intensity of training of every session will be monitored using polar heart rate monitors (F6 or RCX5, Polar Oy, Finland) and perceived rate of exertion (BORG 6–20) [116]. Target training zone will be 60-80% of maximum heart rate according to the Karvonen formula e.g. 60% training limit = (220 – age) x 0.6 and 80% training limit = (220 – age) x 0.8.

Blood lactate levels will also be measured so as to obtain quantitative measures of training intensity and to ensure all training groups have trained at similar intensities. Samples will be taken during week 12, before training after 15 minutes of rest and 3 minutes after cessation of main strength training session. These will be recorded for each different intensity level of training (barefoot, small and large resistance boots, 45 seconds work per leg). Fingertip blood samples will be taken using safety lancet, normal 21 G with penetration depth 1.8 mm (Sarstedt AG & co, Germany) and collected into 20 μL capillary tubes which are placed in 1-mL hemolyzing solution. Care will be taken to clean skin to avoid contamination from chlorinated pool water. Samples will be analysed using an automatic system (EKF diagnostic, Biosen, Germany) after training.

#### Control group

The control group will be asked to maintain normal physical activity during the intervention period. They will be offered two sham contact sessions consisting of 1 hour of light stretching and relaxation during the 4 month period.

#### Follow up period

After the post intervention measurements all participants will be advised to continue spontaneous physical activity, no other specific instruction will be given to the subject.

#### Ethical considerations

The study was given ethical consent on 30^th^ November 2011 Dnro 19U/2011 from the Ethics Committee of the Central Finland Health Care District. Written informed consent will be obtained from all subjects before their participation in the study. All subjects included have the right to withdraw from the study whenever without needing to provide a reason for withdrawal. The study will be conducted according to good clinical and scientific guidelines and the declaration of Helsinki (2000).

#### Assessment of side effects

Adverse effects or health problems attributable to the testing protocol or interventions exercise protocol will be documented and reported. Following each individual measurement and training session self-reported knee pain will be assessed using a visual analogue scale (VAS 0-100 mm) along with any other physical symptoms such as pain elsewhere than knee, stiffness and general fatigue. All subjects will have medical insurance and have access to the attending medical physician free of charge throughout the 4 month intervention and 12 month follow up period.

#### Statistical analysis

All analyses will be based on both intention-to-treat and dose related principles. Statistical analyses will be performed using statistical software (Stata, release 12.1, StataCorp, College Station, Texas and SPSS Version 19, IBM Corporation).

## Discussion

This paper describes the rationale and design of a randomised control trial investigating the effect a progressive aquatic resistance training program will have on patellofemoral and tibiofemoral cartilage, properties of bone and body composition and physical function in post-menopausal women with mild knee osteoarthritis.

Exercise is one of the main non-pharmaceutical treatments recommended in the management of lower limb OA [24-26,28,29]. It is presumed that training in an aquatic environment has benefits for persons suffering from lower limb OA, however exact content and intensity of optimal training remain unclear [22]. For persons with knee and/or hip OA there is strong evidence to suggest aquatic exercise can cause a small but significant reduction in pain [30,33,34,36,38,123-127], improves self-assessed and measured function with a small to moderate effect size [33-36,38,123-125,127-130]. In addition, there is moderate evidence to show that aquatic exercise can cause a small but significant improvement in aerobic fitness [33,35,127,129,131]. Further there is limited data to suggest aquatic exercise can increase lower limb strength [33-39] and improve balance and decrease risk of falling [36,39,40]. Intensities of interventions in previously studies may not have been high enough to produce large changes in muscle strength and cardiovascular fitness but reporting of exercise programs used are in most cases incomplete. There are few studies investigating the effect of a progressive resistance program using specifically designed resistance equipment to manage symptoms associated with knee OA even though there is accumulating evidence to suggest it can be effective in improving neuromuscular function [117,118,120-122]. Also, there is some evidence to suggest water based exercise can either maintain [132] or slightly improve the properties of bone as measured with DXA [133]. However these are of low quality evidence and further research is required to validate the findings.

Both dGEMRIC [63,134] and T2 relaxation MRI [7,72,135] can distinguish between normal and OA cartilage. These techniques have been shown to be sensitive enough to demonstrate acute changes in human cartilage dGEMRIC [42,67] and T2-relaxation times [42,136]. These methods are therefore suitable for use in our study, and it is known that correct biomechanical loading of cartilage is important in maintaining cartilage health whereas obesity and trauma are risk factors for the development of OA [1]. Although there is evidence to show that biochemical characteristics of cartilage can be negatively affected with changes after periods of joint immobilization [137,138] and non-weight bearing [136]. No evidence exists to show the impact of an intensive non-impact exercise on cartilage.

As far as we know there have been no publications investigating the effect of aquatic exercise on cartilage and properties of bone in persons with knee OA. The aim of this study is to use repetitive aquatic resistance program with high intensity and repetition to discover what effects non-impact training has on knee cartilage, properties of bone and physical function. The information gained will help improve our understanding of the effects of exercise on the biochemical properties of cartilage and improve prescription of aquatic exercises in the management of OA.

## Abbreviations

OA: Osteoarthritis; ROM: Range of Motion; pQCT: Peripheral quantitative computed tomography; DXA: Dual-energy X-ray absorption; WOMAC: Western Ontario and McMaster Universities Osteoarthritis Index; KOOS: Knees injury and Osteoarthritis Outcome Score; CV: Coefficient of Variation; VAS: Visual Analogue Scale; RPE: Rate of Perceived Exertion; MRI: Magnetic Resonance Imaging; dGEMRIC: Delayed Gadolinium-Enhance Magnetic Resonance Imaging of Cartilage; FOV: Field of View; TR: Repetition Time; TE: Echo Times; ETL: Echo Train Length; GAG: Glycosaminoglycan; BMD: Bone Mineral Density; BSI: Bone Strength Index; BMC: Bone Mineral Content; ToD: Total Density; TrD: Trabecular density; ToA: Total Area; TrA: Trabecular Area; aBMD: Areal Bone Mineral Density; BMC: Bone Mineral Content; AHA: Advance Hip structure Analysis; HAL: Hip Axis Length; CSA: Cross Sectional Area; CSMI: Cross Sectional Moment of Inertia (CSMI); FSI: Femoral neck Strength Index; BMI: Body Mass Index; CMJ: Counter Movement Jump; ECG: Echocardiogram.

## Competing interests

All authors declare that they have no competing interests.

## Authors’ contributions

All authors were involved in the conception of the study plan and design as well as critically revising the draft manuscript for important intellectual content. All authors approved the final version to be published. BW, MM, JM and AHeinonen drafted the manuscript.

## Pre-publication history

The pre-publication history for this paper can be accessed here:

http://www.biomedcentral.com/1471-2474/14/82/prepub
